# Equitable Access to Telehealth and Other Services for Deaf People During the COVID-19 Pandemic

**DOI:** 10.1089/heq.2022.0115

**Published:** 2023-02-28

**Authors:** Christopher J. Moreland, Sowmya R. Rao, Katja Jacobs, Poorna Kushalnagar

**Affiliations:** ^1^ Center for Deaf Health Equity, Gallaudet University, Washington, District of Columbia, USA.; ^2^ Dell Medical School at the University of Texas, Austin, Texas, USA.; ^3^ Boston University School of Public Health, Boston, Massachusetts, USA.

**Keywords:** deaf, equity, access, telehealth

## Abstract

**Introduction:**

Deaf people who use American Sign Language (ASL) with low self-perceived ability to understand spoken information face inequitable access to health care due to systemic barriers.

**Methods:**

We conducted interviews with 266 deaf ASL users at baseline (May–Aug 2020) and 244 deaf ASL users at follow-up (3 months). Questions addressed (1) access to interpretation during in-person visits; (2) whether they visited clinics (3) or emergency departments (EDs); and (4) telehealth use. Analyses involved univariate and multivariable logistic regressions across levels of perceived ability to understand spoken language.

**Results:**

Less than a third were aged >65 (22.8%); Black, Indigenous, People of Color (28.6%), or LGBTQ+ (31.1%); and had no college degree (30.6%). More respondents reported outpatient visits at follow-up (63.9%) than at baseline (42.3%). Ten more respondents reported going to urgent care or an ED at follow-up than at baseline. At follow-up interviews, 57% of deaf ASL respondents with high perceived ability to understand spoken language reported receiving interpretation at clinic visits compared to 32% of ASL respondents with low perceived ability to understand spoken language (*p*<0.01). Telehealth and ED visits showed no between-group differences for low versus high perceived ability to understand spoken language.

**Discussion:**

Our study is the first to explore deaf ASL users' access to telehealth and outpatient encounters over time during the pandemic. The U.S. health care system is designed for people who have high perceived ability to understand spoken information. Systemic access to health care, including telehealth and clinics, must be made consistently equitable for deaf people who require accessible communication.

## Introduction

When the COVID-19 pandemic struck in 2020, U.S. health care delivery shifted massively, including increased dependence on virtual telecare and changing encounter policies to emphasize telehealth. For deaf people who use visual communication (e.g., American Sign Language [ASL]), these shifts require adjustments in accommodations like video remote interpreting (VRI) for patients, which can bring benefits, including convenience, reduced likelihood of viral exposure, and increased accessibility.^1^ Such changes also bring new barriers as a result of inaccessible infrastructure for deaf patients through telehealth and in-person visits. The intersection of intrinsic (e.g., age, communication skills, resilience) and extrinsic (e.g., access to information, social support) factors has the potential to contribute to lower quality of life and negative health outcomes for deaf people.^2–5^ For example, deaf Americans who are less fluent in English are more likely to have low health literacy and are at a higher risk for health disparities.^6,7^

Deaf people have reported significant difficulty accessing COVID-specific information in a format that they can access and understand (e.g., ASL or captions) and not trusting that information.^5,8^ No single solution makes telehealth and in-person visits (e.g., hospitals and emergency departments [EDs]) accessible, as communication needs among deaf people vary.^9,10^ From a systemic framework, health care settings that create barriers to access can result in low return visits and low adherence screening rates.^11^ James et al. developed a conceptual model indicating how these barriers and other factors at multiple levels combine to obstruct deaf patients' access to equitable ED utilization, reinforced by interviews about deaf and hard of hearing (DHH) patients' ED experiences.^12,13^

While in-person outpatient visits decreased by 30% from January 1 to June 10 among over 16 million patients, telehealth visits increased by 2013% as a result of the pandemic.^14^ Telehealth has become an increasingly widespread tool for providing services among health care providers following the COVID-19 outbreak. However, with limited consideration for telehealth platform accessibility, deaf people's ability to receive quality care is limited.^14–16^ In a study during the pandemic, over half of deaf telehealth users reported difficulty with communication.^17^

In 2018, ∼20% of older patients were found to be unprepared for telephone visits with their health care providers because of “difficulty hearing, difficulty communicating, or dementia.”^18^ Within the subgroup of older patients, those who were men, unmarried, Black or Hispanic, or had less education or lower income were similarly less prepared.^19^ When considering the deaf, ASL-using community, these sociodemographic characteristics coupled with inaccessible telehealth services for deaf people on the provider's end may exacerbate inequities in quality care.^15^

Health care facilities and services—such as EDs or clinics—have become even less accessible to people with disabilities during the COVID-19 pandemic.^16^ Similarly, these barriers can be reflected in deaf populations due to unavailability of in-person interpreters or low-quality VRI. Providing interpretation services for people with limited English proficiency can improve access to and utilization of health care services.^19^ For deaf people who prefer using interpreters, the quality of health care is impacted when medically trained interpreters are not present.^20^ In a 2019 study, only 41% of participants were satisfied with VRI technology.^21^

Transformation in the way people receive virtual and in-person health care during the COVID-19 pandemic has exacerbated some barriers to care already observed before the pandemic for deaf people.^22^ No data exist on whether those barriers to health care communication via telehealth or in-person might improve over time as health care systems adapt to new pandemic-driven protocols. To achieve health equity in the U.S. health system, the communication needs of deaf people must be considered as delivery methods of health care continue to evolve during the pandemic.

No research to date has investigated the impact of COVID-19 pandemic on the access to U.S. health care for deaf people or how their access to care (e.g., via interpretation services) might change during the pandemic. The study aims are to (1) evaluate changes in access to telehealth or in-person care from baseline to the 3-month follow-up and (2) identify subgroups of deaf people who continue to experience barriers to telehealth or in-person care. We hypothesized that deaf people would report improved access to care over time and, in particular, that those with high (vs. low) perceived ability to understand spoken language would experience significantly improved access.

## Methods

### Recruitment and interview protocol

In brief, following Institutional Review Board approval, research staff recruited DHH ASL speakers who gave permission to be contacted through the Center's recruitment database pool between April 17, 2020 and May 1, 2020. Only those who used ASL and were born DHH or became DHH before 13 years old were included because this group was identified as a medically underserved group. After the participant viewed the study information in ASL and English online, the participant was directed to a page where they could choose to provide consent to voluntarily participate or decline to complete an online demographics survey and participate in a virtual interview with research staff fluent in ASL.

The demographics survey included age, gender, education, race/ethnicity, and perceived communication ability (ability to understand spoken information one on one in a quiet setting). Baseline interviews about health care access were conducted virtually between May 2020 and August 2020. Follow-up virtual interviews occurred September-November 2020, 3 months after the individual's baseline interview.

Interviews were, with consent, recorded via Zoom; recordings were destroyed after data entry and transcriptions completed by an ASL-English bilingual staff member. For those who were unable to access Zoom, a videophone interview was conducted as an alternative and the interviewer took detailed notes during the interview. Each baseline interview took ∼30 min. Participants received a $20 gift card.

Participants who completed the baseline interview received an invitation to participate in a 3-month follow-up videoconference interview, following similar procedures to the baseline interviews as described above. Participants received another $20 gift card.

### Interview design

A sequential exploratory design was used to first gather qualitative data, which were then converted into a quantitative measure that was used for follow-up data collection and analysis. Baseline interview questions were multiple choice or open-ended. For example, if a person said yes to a question about suspicions of having been infected by COVID-19 without testing positive, a follow-up open-ended question allowed free-text expansion. All open-ended responses in ASL were transcribed by a bilingual research assistant and reviewed by the principal investigator PK.

Baseline interview transcriptions were analyzed for recurrent responses to each question to identify common themes. Recurrent responses were coded into multiple response categories and used as multiple choices in close-ended questions during the follow-up interview three months later. Box 1 provides an example of a question converted from open-ended (baseline interview) to multiple-choice (follow-up interview).

### Statistical analyses

Only respondents who participated in both baseline and follow-up interviews were included in the analysis. Perceived ability to understand spoken language was defined by one's self-reported ability to comprehend spoken information by listening and/or speech-reading in a quiet room. This perceived ability question was used in lieu of audiological hearing level.^23^ Perceived ability to understand spoken language was dichotomized into high (understand all/most) versus low (understand some/little/none). Those who reported understanding most or all of what was spoken were classified as high, while those who reported understanding some to none of what was spoken were classified as low; this approach has been described elsewhere.^23^

**Box 1.**

**Baseline**

1. When doctors and nurses are concerned about the coronavirus, they wear special equipment, including a gown, gloves, mask, and eye protection. Interpreters can also wear this. How did their wearing this equipment affect your communication experience in the hospital? [Open-ended question]

**Follow-up**

2. How did the providers' and interpreter's personal protective equipment (PPE) affect your communication experience?
  a. Blocked facial expressions, body language, or lipreading
  b. Provider or interpreter pulled down mask/clear mask which helped
  c. Clear mask didn't help (foggy); I didn't want them to pull down mask
  d. No impact on communication

Questions for participants addressed four outcomes of interest: (1) access to ASL/English interpretation to communicate with a health care provider during an in-person clinic visit; (2) clinic visits during the pandemic; (3) ED or UC site visits during the pandemic; and (4) using telehealth to visit a health care provider for the respondent or their loved ones. The interview script is provided in an Appendix A1. The questions were developed and reviewed iteratively by study investigators PK, CM.

We obtained summary statistics (proportions, means, standard deviations) for all variables. We conducted bivariate analyses to assess the significance of the differences in the distribution of the characteristics and other variables by perceived ability using chi-square tests. Further, we conducted bivariate analyses to assess the significance of the differences in the distribution of the variables indicating telehealth accessibility, and both medical and clinical visits between baseline and follow-up with McNemar's test.

Adjusted odds ratios (AORs) and confidence intervals (95% CIs) were obtained from four separate Generalized Estimating Equation multivariable logistic regression models accounting for the clustering of observations within subjects to assess whether the outcomes varied over time and across perceived ability to understand spoken language. Each model included time (baseline, follow-up), dichotomized perceived ability to understand spoken language (low vs. high), an interaction of time and perceived ability to understand spoken language, age (continuous), preferred language (signed, both signed/spoken), income category (lower, middle/upper), and race (white, Black, Indigenous, People of Color [BIPOC]). In our study, BIPOC people include those who are African American, Asian, or Hispanic. All analyses were conducted in SAS 9.4 (SAS Institute, Cary, NC) and a two-sided *p*<0.05 was considered significant.

## Results

Of 266 respondents to the baseline survey, 244 (93%) responded to the follow-up interviews; baseline characteristics for those 244 respondents are displayed in Table 1, both overall and by perceived ability to follow spoken information through speech-reading and/or listening. Overall, less than half of the respondents were older than 65 years (22.8%); identified as BIPOC (28.6%) or (31.1%); or had high school or some college education (30.6%), higher income (17.5%), no health insurance (6.4%), no employment (42.5%), obesity (28.8%), or high perceived ability to comprehend spoken information (27.7%).

**Table 1.** Distribution of Respondent Characteristics at Baseline—Overall and by Perceived Ability to Understand Spoken Language

Variable	Overall (*N*=244)	Perceived ability to understand spoken language		*p* ^a^
		Understand all/most (*N*=61)	Understand some/little/none (*N*=159)	
Respondent characteristics	*N*^b^ (Col%)	*N*^b^ (Row%)		
Age in years				
18–34	49 (20.3)	16 (38.1)	26 (61.9)	0.03
35–49	64 (26.6)	20 (32.3)	42 (67.7)	
50–64	73 (30.3)	18 (26.9)	49 (73.1)	
65+	55 (22.8)	6 (12.8)	41 (87.2)	
Race				
African American/Black	13 (5.4)	5 (45.5)	6 (54.5)	0.46
Asian/other	26 (10.8)	8 (33.3)	16 (66.7)	
Hispanic	30 (12.4)	7 (24.1)	22 (75.9)	
White	172 (71.4)	41 (26.3)	115 (73.7)	
Education				
High school degree	30 (12.4)	7 (28.0)	18 (72.0)	0.97
Some college	44 (18.2)	11 (28.9)	27 (71.1)	
College graduate	168 (69.4)	43 (27.4)	114 (72.6)	
Sexual orientation				
Straight	162 (68.9)	36 (24.3)	112 (75.7)	0.32
LGBQA+	73 (31.1)	21 (31.3)	46 (68.7)	
Preferred language				
Signed language	113 (50.9)	18 (16.1)	94 (83.9)	<0.0001
Both signed and spoken language	109 (49.1)	43 (39.8)	65 (60.2)	
Marital status				
Married/living with a partner	124 (51.0)	28 (23.9)	89 (76.1)	0.23
Divorced/widowed/separated/never married	119 (49.0)	33 (32.0)	70 (68.0)	
Income range				
Lower	36 (35.0)	12 (33.3)	24 (66.7)	0.07
Middle	49 (47.6)	14 (28.6)	35 (71.4)	
Upper	18 (17.5)	1 (5.6)	17 (94.4)	
Have health insurance				
No	15 (6.4)	6 (46.2)	7 (53.8)	0.19
Yes	219 (93.6)	52 (26.0)	148 (74.0)	
Any lifetime medical diagnosis				
No	26 (15.0)	11 (42.3)	15 (57.7)	0.05
Yes	147 (85.0)	30 (21.9)	107 (78.1)	
Occupation status				
Employed	132 (54.3)	39 (32.0)	83 (68.0)	0.13
Unemployed/homemaker/student/retired/disabled	111 (45.7)	22 (22.4)	76 (77.6)	
Self-reported body mass index				
<25.0	35 (33.7)	10 (28.6)	25 (71.4)	0.85
25.0–29.9	39 (37.5)	9 (23.1)	30 (76.9)	
≥30.0	30 (28.8)	8 (26.7)	22 (73.3)	
Health care experience: baseline	*N* (Col %)			
Visit any clinic or doctor's office in person during the pandemic				
No	124 (57.7)	33 (63.5)	81 (57.4)	0.51
Yes	91 (42.3)	19 (36.5)	60 (42.6)	
Got an interpreter when needed to communicate with a health care provider during this clinic or in-person visit at a doctor's office				
No	50 (78.1)	9 (69.2)	35 (79.5)	0.47
Yes	14 (21.9)	4 (30.8)	9 (20.5)	
Go to an emergency room or urgent care during the pandemic				
No	213 (88.8)	54 (90.0)	137 (87.8)	0.81
Yes	27 (11.3)	6 (10.0)	19 (12.2)	
Use telehealth to visit you or your loved ones' (e.g., parents, children) health care provider				
No	118(49.2)	33 (55.0)	72 (46.2)	0.29
Yes	122(50.8)	27 (45.0)	84 (53.8)	
Health care experience: follow-up	N (Col %)			
Visit any clinic or doctor's office in person during the pandemic				
No	68 (30.5)	14 (26.4)	49 (32.9)	0.49
Yes	155 (69.5)	39 (73.6)	100 (67.1)	
Got an interpreter when needed to communicate with a health care provider during this clinic or in-person visit at a doctor's office				
No	90 (60.0)	16 (43.2)	66 (68.0)	0.01
Yes	60 (40.0)	21 (56.8)	31 (32.0)	
Go to an emergency room or urgent care during the pandemic				
No	186 (83.4)	46 (86.8)	124 (83.2)	0.66
Yes	37 (16.6)	7 (13.2)	25 (16.8)	
Use telehealth to visit you or your loved ones' (e.g., parents, children) health care provider				
No	186 (83.4)	26 (49.1)	77 (51.7)	0.75
Yes	37 (16.6)	27 (50.9)	72 (48.3)	
Did the provider who you saw the most during the pandemic do a good job of accommodating you as a deaf patient (e.g., telehealth, video relay interpreting)—follow-up				
No	16 (9.9)	6 (15.8)	6 (5.6)	0.08
Yes	145 (90.1)	32 (84.2)	101 (94.4)	
Health care provider tell you that you had or were diagnosed with COVID-19—follow-up				
No	220 (98.7)	52 (98.1)	148 (99.3)	0.46
Yes	3 (1.3)	1 (1.9)	1 (0.7)	

^a^ Based on two-sided chi-square.

^b^ Might not add up to the total due to missing values.

As shown in Table 1, deaf ASL users with low and high perceived ability to understand spoken information were comparable for having used telehealth, clinic, or ED services at both baseline and follow-up interviews. Significant group differences were observed for getting an interpreter when needed to communicate with a health care provider during in-person clinic visits. At the follow-up interview, about 57% of deaf ASL respondents with high perceived ability to understand spoken language stated that they received an interpreter at their clinic visits compared to only 32% of deaf ASL respondents with low perceived ability to understand spoken language (*p*<0.01).

For the 223 who answered all telehealth questions at follow-up, Table 2 displays the response changes between baseline and follow-up for questions on telehealth accessibility, medical/clinic visits, and participant perception of telehealth communication. At baseline, 112 participants used telehealth while 101 did so at follow-up. At baseline, of 81 who visited a clinic during the pandemic, only 4 used a speech-to-text recognition app and 9 got an interpreter when needed, while 38 requested an interpreter but did not get one. Of the 26 who visited an ED or urgent care (UC), 2 used a live transcription app and 12 needed an interpreter.

**Table 2.** Distribution of Telehealth and In-person Accessibility Variables at Baseline and Follow-up

Variable	Baseline	Follow-up		*p* ^a^
		Yes	No	
	*N*^b^ (Col%)	*N*^b^ (Row%)		
COVID-19 knowledge and action				
Even though your health care provider said that you did not have COVID-19, think you might have had the coronavirus				
Yes	48	24 (50.0)	24 (50.0)	0.21
No	167	16 (9.6)	151 (90.4)	
Emergency department and urgent care visits				
Go to an emergency room or urgent care during the pandemic				
Yes	26	16 (61.5)	10 (38.5)	0.07
No	193	20 (10.4)	173 (89.6)	
Need an interpreter during this emergency room or urgent care visit				
Yes	12	12 (100.0)	0 (0.0)	0.32
No	2	1 (50.0)	1 (50.0)	
Interpreter provided in person or through VRI during this emergency room or urgent care visit				
On Site	3	0 (0.0)	3 (100.0)	0.32
VRI	8	1 (12.5)	7 (87.5)	
Use a live transcription app that translates speech to text during your emergency service or urgent care visit				
Yes	2	2 (100.0)	0 (0.0)	0.16
No	13	2 (15.4)	11 (84.6)	
Hospital visits				
Stay in the hospital for a few hours or >1 day				
Yes	16	4 (25.0)	12 (75.0)	0.21
No	203	19 (9.4)	184 (90.6)	
Clinic visits				
Visit any clinic or doctor's office in person during the pandemic				
Yes	81	62 (76.5)	19 (23.5)	<0.0001
No	119	76 (63.9)	43 (36.1)	
Need an interpreter during this clinic or in-person visit at a doctor's office				
Yes	47	38 (80.9)	9 (19.1)	0.81
No	15	8 (53.3)	7 (46.7)	
Got an interpreter when needed to communicate with a health care provider during this clinic or in-person visit at a doctor's office				
Yes	9	6 (66.6)	3 (33.3)	0.21
No	38	7 (18.4)	31 (81.6)	
Interpreter provided in person or through VRI during this clinic or in-person visit at a doctor's office				
On Site	19	15 (78.9)	4 (21.1)	0.74
VRI	16	5 (31.3)	11 (68.8)	
Use a live transcription app that translates speech into text during clinic or in-person visit at a doctor's office				
Yes	4	1 (25.0)	3 (75.0)	0.21
No	57	7 (12.3)	50 (87.7)	
Telehealth				
Use telehealth to visit your or your loved ones' (e.g parents, children) health care provider				
Yes	112	74 (66.1)	38 (33.9)	0.17
No	107	27 (25.2)	80 (74.8)	
Able to see interpreter clearly during the visit				
Yes	42	37 (88.1)	5 (11.9)	0.03
No	0	0 (0.0)	0 (0.0)	
Able to see the provider clearly during the visit				
Yes	18	14 (77.8)	4 (22.2)	0.74
No	11	5 (45.5)	6 (54.5)	
Difficult to set up a telehealth appointment				
Yes	12	8 (66.7)	4 (33.3)	0.53
No	12	6 (50.0)	6 (50.0)	
Patient-centered communication				
Did the provider who you saw the most during the pandemic do a good job of accommodating you as a deaf patient (e.g., telehealth, VRI)				
Yes	5	5 (100.0)	0 (0.0)	0.03
No	7	5 (71.4)	2 (28.6)	
Needs assessment				
Know any deaf person who is experiencing mental health issues because of the COVID-19 pandemic				
Yes	113	95 (84.1)	18 (15.9)	0.43
No	57	23 (40.4)	34 (59.6)	
Did the deaf person you know who experienced mental health issues because of the COVID-19 pandemic get access to mental health services				
Yes	5	2 (40.0)	3 (60.0)	0.03
No	16	11 (68.8)	5 (31.3)	

^a^ Based on McNemar's test.

^b^ Only among those that responded to both the baseline and follow-up.

VRI, video remote interpreting.

There were some items indicating significant changes from baseline to follow-up. However, for most survey items, responses did not change significantly. More respondents (*n*=155; 69.5%) reported an outpatient visit at follow-up than at baseline (*n*=91; 42.3%); 76 of 119 (63.9%) who reported not having a visit at baseline reported a visit at follow-up (*p*<0.0001).

Eleven (68.8%) of 16 respondents, who stated that they knew deaf people personally with mental health issues due to the pandemic who did not get access to mental health services at baseline, reported that those people received access at follow-up: an increase from 23.8% (*n*=5) at baseline to 52.4% (*n*=11) at follow-up (*p*=0.03). Ten more respondents reported going to UC or an ED at follow-up than at baseline. Of seven who reported poor accommodation as a deaf person by their most frequent provider at baseline, five (71.4%) reported accommodation at follow-up.

Although not statistically significant, multivariable regression results (Table 3) indicated that between baseline and follow-up, those with high perceived ability to understand spoken language (vs. Low perceived ability) were more likely to (1) get an interpreter when needed to communicate with a health care provider during an in-person clinic visit (AOR [95% CI]: 1.57 [0.62–3.96]; *p*=0.34); (2) visit any clinic during the pandemic (2.55 [0.56–11.57]; *p*=0.22); (3) go to an ED or UC during the pandemic (1.05 [0.25–4.34]; *p*=0.95); and (4) use telehealth visits with providers for themselves or their loved ones (1.63 [0.55–4.88]; *p*=0.38) after adjusting for age, preferred language, income category, and race.

**Table 3.** Results from Multivariable Logistic Regressions Assessing the Relationship of the Characteristics with Outcomes

Variables	Outcomes			
	Got an interpreter when needed to communicate with a health care provider during this clinic or in-person visit at a Doctor's office	Visit any clinic or doctor's office in person during the pandemic	Go to an emergency room or urgent care during the pandemic	Use telehealth to visit your or your loved ones' (e.g., parents, children) health care provider
Adjusted odds ratio (95% confidence intervals)^a^; *p*-values				
Functional hearing: understand most/all vs. understand some/little/none	2.02 (0.51–7.97); 0.31	0.42 (0.14–1.23); 0.11	0.92 (0.22–3.85); 0.91	0.27 (0.10–0.74); 0.01
Interview time: follow-up vs. baseline	0.77 (0.33–1.84); 0.56	4.43 (2.23–8.82); <0.0001	1.40 (0.68–2.87); 0.35	0.87 (0.47–1.58); 0.64
Interaction: functional hearing: understand all/most and Time=follow-up	1.57 (0.62–3.96); 0.34	2.55 (0.56–11.57); 0.22	1.05 (0.25–4.34); 0.95	1.63 (0.55–4.88); 0.38
Age	0.99 (0.96–1.03); 0.72	1.02 (1.00–1.05); 0.06	1.00 (0.97–1.03); 0.86	0.99 (0.96–1.01); 0.28
Preferred language: both signed/spoken vs. signed	1.21 (0.41–3.52); 0.73	1.10 (0.55–2.16); 0.79	1.52 (0.58–3.99); 0.40	2.38 (1.15–4.93); 0.02
Income: middle/upper vs. lower	0.60 (0.20–1.80); 0.36	0.79 (0.37–1.65); 0.53	2.57 (0.82–8.11); 0.11	1.29 (0.63–2.64); 0.48
Race: African American/Asian/Hispanic/Other vs. White	—	1.52 (0.70–3.29); 0.29	1.21 (0.43–3.45); 0.71	0.75 (0.36–1.57); 0.45

^a^ Based on multivariable models that included all variables showed in the table. Due to small cell sizes, race was not included in the model to assess the relationship of characteristics with “Got an interpreter when needed to communicate with a healthcare provider during this clinic or in-person visit at a Doctor's office.”

## Discussion

Our study is the first to explore deaf ASL users' access to telehealth, outpatient, and inpatient encounters over 3 months during the pandemic; study findings suggest that, reassuringly, participants overall reported more clinic and other encounters at follow-up compared to baseline. This trend reflects another study in that outpatient visits stably returned to prepandemic levels as of July through August, 2020.^24^ However, this increase in clinic visits was not paralleled by equitable accommodation services in our study.

Those with low perceived ability to understand spoken language reported at follow-up being significantly less likely to receive interpretation services at a clinic than those who understood most or all spoken language (Table 1); in addition, individuals with greater perceived ability to understand spoken language appeared more likely to visit clinics, get an interpreter for such visits, and use telehealth based on multivariable analysis results. Health care access disparities *within* the deaf community thus may have been unmasked during the pandemic.

A number of mechanisms may explain this disparity. The process of requesting interpreters for clinics in the context of COVID-19 precautions may have been easier for those with high perceived ability to understand spoken language (e.g., ability to navigate and follow instructions to request interpreters; ability to communicate through spoken or written English, which accommodates the dominant language used by most health care staff). Alternatively, interpretation service quality in the context of face masking may have benefited those with high perceived ability who might listen to spoken English in combination with sign language interpretation. Other pathways to more effective health care communication may also appear more accessible to those with high perceived ability to understand spoken language, such as using the spoken word to augment accessing health care systems' interpreter request protocols or even informing providers directly of their need for interpreters.

Several strategies can enhance communication and accommodation access, some of which were informally suggested by study participants. These include identifying and documenting deaf patients' preferred forms of communication (e.g., ASL interpreter, caregiver support, written notes), health care worker training in cultural competency and humility, and transparent facemasks, all of which have the potential to advance communication accessibility for both in-person and telehealth care.^25^

Beyond in-person visits, our findings also show that telehealth visits at baseline as well as follow-up were utilized more often by ASL users with low perceived ability to understand spoken language, compared to those with high perceived ability. This has implications for patient-centered communication which might be compromised in telehealth care, such as through the absence of interpretation services. Research has shown similar levels of satisfaction among patients for both in-person and telehealth visits, with the added benefit of reduced wait, visit, and travel times, as well as cost when telehealth is used.^26,27^ However, we speculate that deaf patients who use ASL are less likely to reap the benefits of telehealth should they face barriers accessing telehealth appointments and communication in the first place.^16,17,19^

Clinicians are also likely to be less trained on accommodating deaf patients through telehealth platforms due to lack of instructions or training by the telehealth supplier.^16^ Future research should examine communication access and inequities for telehealth visits among deaf ASL users as well as enhancing health care team members' familiarity with telehealth features (e.g., chat, captions, ensuring access for third-party interpreters) to support communication accessibility. Future work should also explore what forms of communication take place during telehealth visits and how access can be enhanced.

While telehealth strategies began before and rapidly expanded in utilization during the pandemic, telehealth continues to be implemented in both outpatient and inpatient settings. With this new modality of health care encounters becoming more firmly ensconced, it becomes even more critical that principles of communication equity be implemented consistently across institutions. Some additional suggestions for improvement shared by study participants included allowing third parties on videoconference platforms so that an interpreter can be viewed on the same screen as the clinician and patient. It would also be useful to make captions rapidly available on telehealth platforms as this provides additional communication support for both deaf patients and clinicians during telehealth visits.

Some deaf participants informally expressed frustration with clinicians' not knowing how to use chat features or enable captions that may be available on the platform due to absent instructions. Ensuring that video interpreting platforms are up-to-date reduces the risk of communication barriers and frustration arising between deaf patients and clinicians, especially in the face of clinical urgency.

Our study has limitations. We are unable to describe respondents' accommodation and access experiences before the pandemic. Our sample is also limited to deaf participants who had access to the internet or cellular service that supported video conference interviews; the sample did not include deaf individuals without such resources (e.g., homelessness, uninsured) who are likely at even greater risk for inequitable access to telehealth care. We are also unable to describe the types of facilities where respondents sought care (e.g., academic, private, or federally qualified health care centers) and how this factor might influence existing accommodations or communication experiences.

### Health equity implications

Health care communication challenges for deaf people must be optimized with proactive accommodation planning and access, including telehealth platforms that have become more valuable during the pandemic and are likely here to stay. With the potential for resurgent pandemic concerns or future health care system crises straining communication systems, it remains critical that telehealth and in-person services incorporate interpretation, captioning, and other access measures to ensure equitable treatment for deaf ASL users. Only then can health care systems and clinicians aim to avoid exacerbating and even reduce health care disparities affecting deaf patients.

## Abbreviations Used

AOR: adjusted odds ratios
ASL: American Sign Language
CI: confidence intervals
DHH: deaf and hard of hearing
ED: emergency department
UC: urgent care
VRI: video remote interpreting

## Appendix

### COVID-19 Knowledge and Action

A. Even though your healthcare provider said that you did not have COVID-19, do you think you might have had the coronavirus? If yes, please explain why.

### Emergency Room

A. Did you go to any emergency room or UC during the pandemic?
B. During this emergency room or UC visit, did you need an interpreter?
C. During this emergency room or UC visit, was the interpreter provided in person or through VRI?
D. How well did this interpreter understand you?
E. How well did you understand this interpreter?
F. Please think about your most recent frustrating experience related to communication or accessibility in ED/UC. What was the main reason for this frustration?
G. During your emergency service or UC visit, did you use a live transcription app that captures speech and translates this into text such as Google Live Transcribe, Microsoft Translator, etc. on your smartphone?
H. Describe your experience using an automatic speech recognition (ASR) app during your visit.
I. How did the providers' and interpreter's PPE affect your communication experience?

### Hospital

A. Did you have to stay in the hospital for a few hours or >1 day?
B. During this hospital stay, did you need an interpreter?
C. During this hospital stay, was the interpreter provided in person or through VRI?
D. How well did this interpreter understand you?
E. How well did you understand this interpreter?
F. Please think about your most recent frustrating experience related to communication or accessibility in the hospital. What was the main reason for this frustration?
G. During your hospital stay, did you use a live transcription app that captures speech and translates this into text such as Google Live Transcribe, Microsoft Translator, etc. on your smartphone?
H. Describe your experience using an ASR app during your visit.
I. How did the providers' and interpreter's PPE affect your communication experience?

### Clinic

A. Did you visit any clinic or doctor's office in person during the pandemic?
B. During this clinic or in person visit at a doctor's office, did you need an interpreter?
C. During this clinic or in person visit at a doctor's office, was the interpreter provided in person or through VRI?
D. How well did this interpreter understand you?
E. How well did you understand this interpreter?
F. Please think about your most recent frustrating experience related to communication or accessibility at a clinic or doctor's office. What was the main reason for this frustration?
G. During your clinic or in-person visit at a doctor's office, did you use a live transcription app that captures speech and translates this into text such as Google Live Transcribe, Microsoft Translator, etc. on your smartphone?
H. Describe your experience using an ASR app during your visit.
I. How did the providers' and interpreter's PPE affect your communication experience?

### Telehealth

A. Did you use telehealth to visit your or your loved ones' (e.g., parents, children) healthcare provider?
B. How did you use your telehealth service to communicate with your healthcare professional?
C. Did you get an interpreter for your telehealth appointment?
D. What was the reason for not getting an interpreter for your telehealth visit?
E. Were you able to see the interpreter clearly during the visit?
F. How well did this interpreter understand you?
G. How well did you understand this interpreter?
H. What were the reasons for not being able to understand the interpreter or vice versa?
I. Were you able to see the provider clearly during the visit?
J. What were the reasons for not being able to see the provider?
K. How did you feel about communicating with your provider through telehealth? Please also include your experience with communicating through emails or health portals.
L. Was it difficult to set up a telehealth appointment?
M. How did you feel about communicating with your provider through telehealth?
N. After COVID-19 pandemic ends and doctors start doing in-person visits again, would you like to keep doing telehealth service as an option?

### Patient-Centered Communication

A. Please think about the provider who you saw the most during the pandemic. Did this provider do a good job of accommodating you as a deaf patient (e.g., telehealth, VRI)?

### Needs assessment

A. Do you know any deaf person who is experiencing mental health issues because of the COVID-19 pandemic?
B. You said that you know a deaf person who experienced mental health issues because of the COVID-19 pandemic. Did this person get access to mental health services?

### Demographics

A. How old are you?
B. What is the highest grade or level of schooling you completed?
C. What is your race? Select all that apply.
D. Do you have any kind of health care coverage, including insurance, prepaid plans such as health maintenance organizations or government plans such as Medicare?

### Deaf Demographics

A. If a person speaks to you in a quiet room, how much can you understand what the person says?
